# Resilience and burden in family caregivers of patients with severe mental disorders

**DOI:** 10.1192/j.eurpsy.2024.1260

**Published:** 2024-08-27

**Authors:** R. Masmoudi, W. Abid, E. Ben Salem, R. Ouali, I. Feki, I. Baati, J. Masmoudi

**Affiliations:** psychiatry A department, hedi chaker university hospital, sfax, Tunisia

## Abstract

**Introduction:**

Family caregivers of patients suffering from severe psychiatric disorders may present with health problems, lower quality of life, and painful emotions, which can seriously compromise their well-being when they do not receive appropriate professional support.

**Objectives:**

The aims of this study were to assess the level of burden and resilience in family caregivers of patients with severe mental disorders and to determine associated factors.

**Methods:**

We conducted a descriptive and analytical cross-sectional study among family caregivers of patients followed at the psychiatry outpatient clinic of the Hedi Chaker University Hospital in Sfax, during the period from February 2022 to July 2022.

We used the Connor–Davidson Resilience Scale (CD-RISC) to assess resilience and the Zarit Burden Inventory to assess the level of burden. Higher scores indicate higher resilience and greater burden.

**Results:**

The sample included 90 family caregivers of patients with severe mental disorders. The average age was 50.68. They were the parents of patients in 40% of cases. Professionally active caregivers accounted for 57.8% of cases. Thirty family caregivers had a somatic disorder history (33.3%).

The median age of patients was 42 years. Ten patients (11.1%) were financially independent. The diagnosis was schizophrenia in 68.9% of cases. The mean duration of illness was 16.23 years. Irregular follow-up was noted in 10 patients (11.1%).

The mean scores of the Zarit Burden scale and the CD-RISC were 41.86± 10.33 and 58.46± 9.18 respectively.

Unemployed caregivers and parents experienced a higher burden (p=0.001, p=0.03 respectively). The level of burden was higher in caregivers taking care of financially dependent patients (p=0.03), with a duration of the disease greater than 15 years (p=0.04), and with irregular follow-up (p=0.008).

A low level of resilience in caregivers was correlated with spousal relationship (p=0.001), cohabitation with the patient (p=0.05), widowhood (p=0.01), low level of education (p=0.02), the presence of a somatic disorder history in the caregivers (p=0.04).

A negative correlation was observed between CD-RISC and Zarit scores (p=0.04; r=-0.21).

**Conclusions:**

Family caregivers of mentally ill patients experienced a significant level of caregiver burden, and it was lower in caregivers with higher levels of resilience. Psycho-educational programs directed toward family caregivers are highly recommended.

**Disclosure of Interest:**

None Declared

